# Optimization of MicroCT Imaging and Blood Vessel Diameter Quantitation of Preclinical Specimen Vasculature with Radiopaque Polymer Injection Medium

**DOI:** 10.1371/journal.pone.0019099

**Published:** 2011-04-18

**Authors:** Sergio X. Vasquez, Feng Gao, Feng Su, Victor Grijalva, John Pope, Bill Martin, Jeroen Stinstra, Matthew Masner, Neha Shah, David M. Weinstein, Robin Farias-Eisner, Srinivasa T. Reddy

**Affiliations:** 1 Numira Biosciences, Salt Lake City, Utah, United States of America; 2 Department of Obstetrics and Gynecology, David Geffen School of Medicine, University of California Los Angeles, Los Angeles, California, United States of America; 3 Department of Medicine, David Geffen School of Medicine, University of California Los Angeles, Los Angeles, California, United States of America; 4 Integrated Laboratory Systems, Durham, North Carolina, United States of America; 5 Department of Molecular and Medical Pharmacology, David Geffen School of Medicine, University of California Los Angeles, Los Angeles, California, United States of America; Brigham & Women's Hospital - Harvard Medical School, United States of America

## Abstract

Vascular networks within a living organism are complex, multi-dimensional, and challenging to image capture. Radio-angiographic studies in live animals require a high level of infrastructure and technical investment in order to administer costly perfusion mediums whose signals metabolize and degrade relatively rapidly, diminishing within a few hours or days. Additionally, live animal specimens must not be subject to long duration scans, which can cause high levels of radiation exposure to the specimen, limiting the quality of images that can be captured. Lastly, despite technological advances in live-animal specimen imaging, it is quite difficult to minimize or prevent movement of a live animal, which can cause motion artifacts in the final data output. It is demonstrated here that through the use of postmortem perfusion protocols of radiopaque silicone polymer mediums and *ex-vivo* organ harvest, it is possible to acquire a high level of vascular signal in preclinical specimens through the use of micro-computed tomographic (microCT) imaging. Additionally, utilizing high-order rendering algorithms, it is possible to further derive vessel morphometrics for qualitative and quantitative analysis.

## Introduction

Silicone polymer injection mediums have been used to visualize vascular networks by tissue clearance and light microscopy for over 40 years [1]. Recently, due to the further development of microCT technology, radiopaque perfusion compounds have become of high interest to researchers hoping to qualify regional therapeutic effect in diseased blood vasculature [2]. The advantages of using microCT to image vasculature include three dimensional imaging and non-destructiveness. Due to proven use in field and low cost, methods were optimized using Microfil (Flow Tech, Carver, MA) compound as the perfusion medium. Owing to the presence of lead chromate in formulation and retention of flexible casting properties when catalyzed and set [3], Microfil provides a number of characteristics amenable to microCT imaging of vascular networks in prepared specimens.

## Materials and Methods

### Small animal preparation and perfusion

All procedures were conducted in accordance with institutional, US and international regulations and standards on animal welfare. Ethical approval was granted by the UCLA animal research committee (animal protocol number 2004-161-13). Five female C57BL/6J mice were isolated from colony prior to perfusion procedure. Specimens were individually heparinized (Sigma Aldrich, H4784) by intraperitoneal injection (50 µl/specimen, 1000 U/ml) and anesthetized by isoflurane inhalation (Attane, 83642) according to approved institutional protocols. Surgical procedures were enacted to prepare the specimen for a postmortem intracardial perfusion. Briefly, a midline incision was made from pubic symphysis to xiphoid process. The diaphragm was severed peripherally to expose the thoracic cavity. The heart was cannulated distally, at the apex of the left ventricle, and the right atrium was severed to provide outflow. To facilitate blood clearance, several volumes of heated saline (37°C) were administered via perfusion pump (LS Economy pump, Cole-Parmer, USA) at a flow rate of 5 ml/min. As the peripheral organs became visibly blanched, the flow rate was lowered to 2 ml/min and the perfusion medium was changed to MicroFil catalyzed at a viscosity appropriate for small vessel filling (2 ml∶5 ml∶225 µL; pigmented compound∶diluent∶curing agent, respectively). At the conclusion of the procedure, specimens were loosely wrapped in aluminum foil and placed in the refrigerator to cure overnight. Individually harvested organs were fixed in 10% neutral buffered formalin and whole specimens were placed in several volumes of 10% neutral buffered formalin.

### MicroCT scanning and parameters

The individual organ specimens were staged in a high-resolution µCT scanner (Scanco µCT40, SCANCO USA, Southeastern, PA) and scanned at 10 µm resolution, 2000 views, 5 frames per view, 300 ms exposure time, 55 kVp, 144 µA. Scan time ranged from 7.4 hours to 17.7 hours. File sizes ranged from 1.5 GB to 6.8 GB. Whole body specimens were staged in a large field of view scanner (Varian Medical Systems, BIR/150/130, Lincolnshire, IL) and scanned at 58 µm resolution, 2880 views, 2 averaged views, 67 ms integration time, 130 kVP, 120 µA. Scan time was 4 hours. File size was 3.5 GB.

### Post-processing and rendering

The microCT generated DICOM files were converted into a file format compatible with GE MicroView (version 2.1.2, GE Healthcare) or AltaViewer (Numira Biosciences Inc., Salt Lake City, UT), which provide both planar views and volume renderings of the samples. A modified version of SCIRun (Scientific Computing Institute, University of Utah, Salt Lake City, UT) was used to generate pseudo-colored volume renderings.

## Results

### Microfil imaged by microCT shows *in-situ* filling of vasculature of preclinical specimen organs in three spatial dimensions

**Figure 1:**
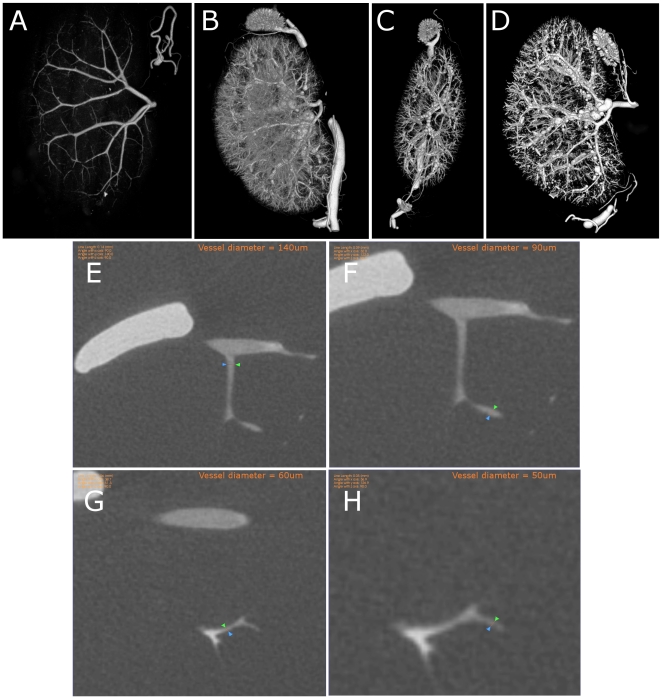
µCT data allows for unrestricted viewing of vascular networks and can be used to perform linear vessel measurements. Manipulations of µCT data sets allow viewing of differing levels of vascular detail as well as unrestricted orientations. (A) Adult mouse kidney with digital threshold set to highlight only major vessels, sagittal view. (B) Adult mouse kidney with digital threshold levels set to highlight fine vessels, sagittal view. (C–D) Adult mouse kidney vasculature viewed in coronal (C) and sagittal (D) orientation. (E–H) Linear length measurements performed on Microfil perfused vessels in the cortical region of the mouse kidney. Vessel diameters of 140 µm (E) are shown progressively, down to 50 µm (H).

After curing, MicroFil perfused organs were scanned at parameters appropriate for deriving fine vascular detail. As shown in Figure 1, the filled lumen of the kidney vasculature is made visible in volume renderings (A–D). At 10 µm spatial resolution, large vessel diameters in excess of 1 mm are captured (data not shown) and fine vasculature (4th order branching in the region of the cortex) can be observed and measured down to 50 µm (E–H). Because region of interest data is of most concern to individual researchers, it is possible to digitally isolate focal areas for preferred observation. Panel A of Figure 1 shows large vessel signal for an adult mouse kidney in a sagittal orientation. Panels B–D show perfused mouse kidneys rendered in various orientations, detailing finer vasculature features, including those of the mouse adrenal gland. Panels E–H show 2-D slice projections of vessels and their measured diameters.

### Microfil image data can be rendered to perform qualitative analysis on specific regions of interest

**Figure 2:**
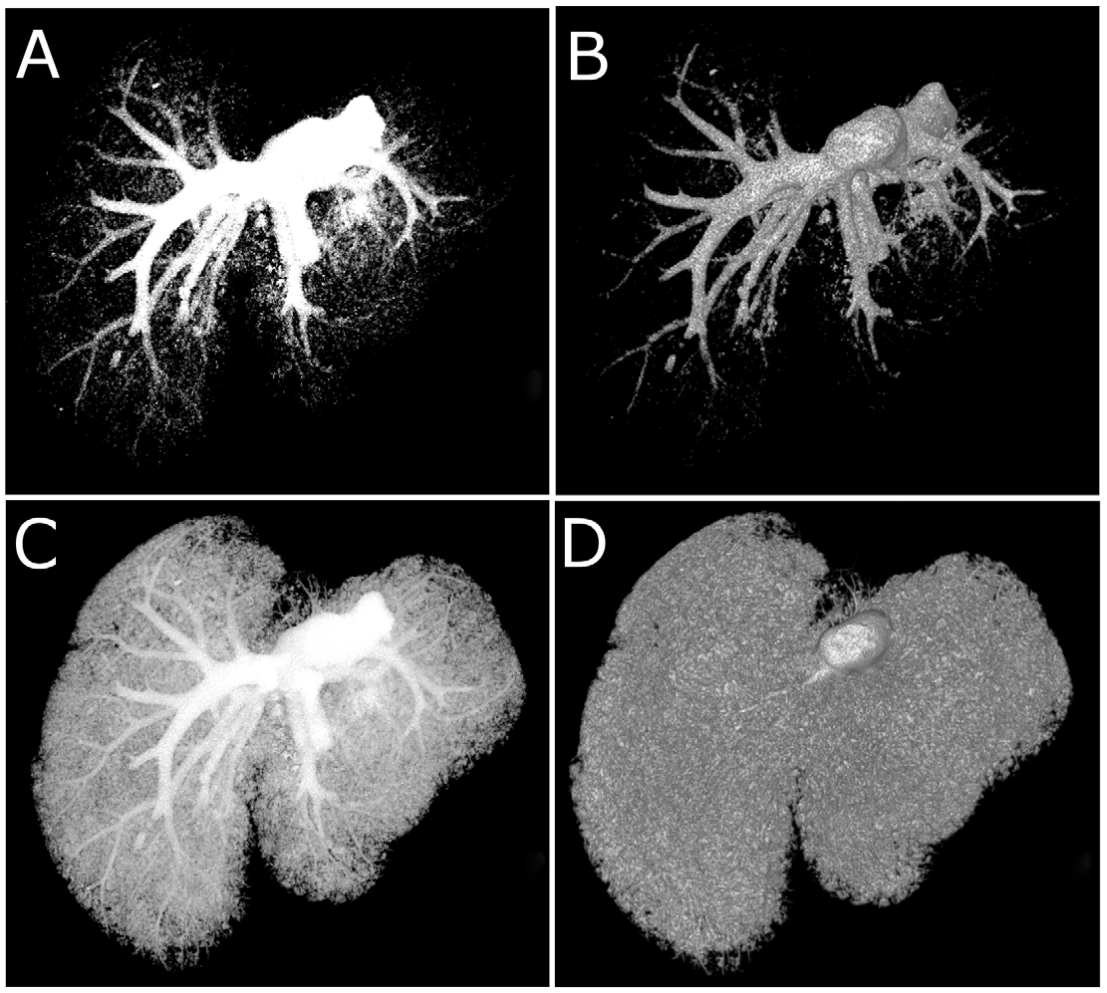
Combined analysis of threshold manipulated MIP and volume rendered data yields greater understanding of vascular networks. All images are of the same Microfil perfused adult mouse liver vasculature. (A) MIP and (B) volume rendered vasculature with thresholds manipulated to highlight the major vessels. (C) MIP and (D) volume rendered vasculature with thresholds manipulated to visualize total vasculature.

MicroFil perfusion methods along with microCT imaging circumvent the need to physically remove anatomical regions of interest and allow non-destructive whole mount imaging of the organ vasculature of interest. In Figure 2, we show that it is possible to isolate hepatic vasculature through digital thresholding while retaining peripheral tissue information. Panel A of Figure 2 depicts a maximum intensity projection (MIP) of the liver vasculature. The highest voxel intensities are depicted in the region of interest, but no depth of field is possible due to signal masking in the hyperattenuating vasculature. An automated volume rendering adjusted to the previous MIP threshold (Panel B, Figure 2) reveals the true vascular orientation. Subtle threshold variations in the MIP rendering derive the image shown in Panel C of Figure 2, depicting the fine peripheral vasculature in relation to the larger diameter vasculature. Volume renderings at the same threshold value depict the whole mount volume of liver vasculature (Panel D).

### Whole mount imaging of specimen vasculature can be achieved through the use of MicroFil perfusion methods

**Figure 3:**
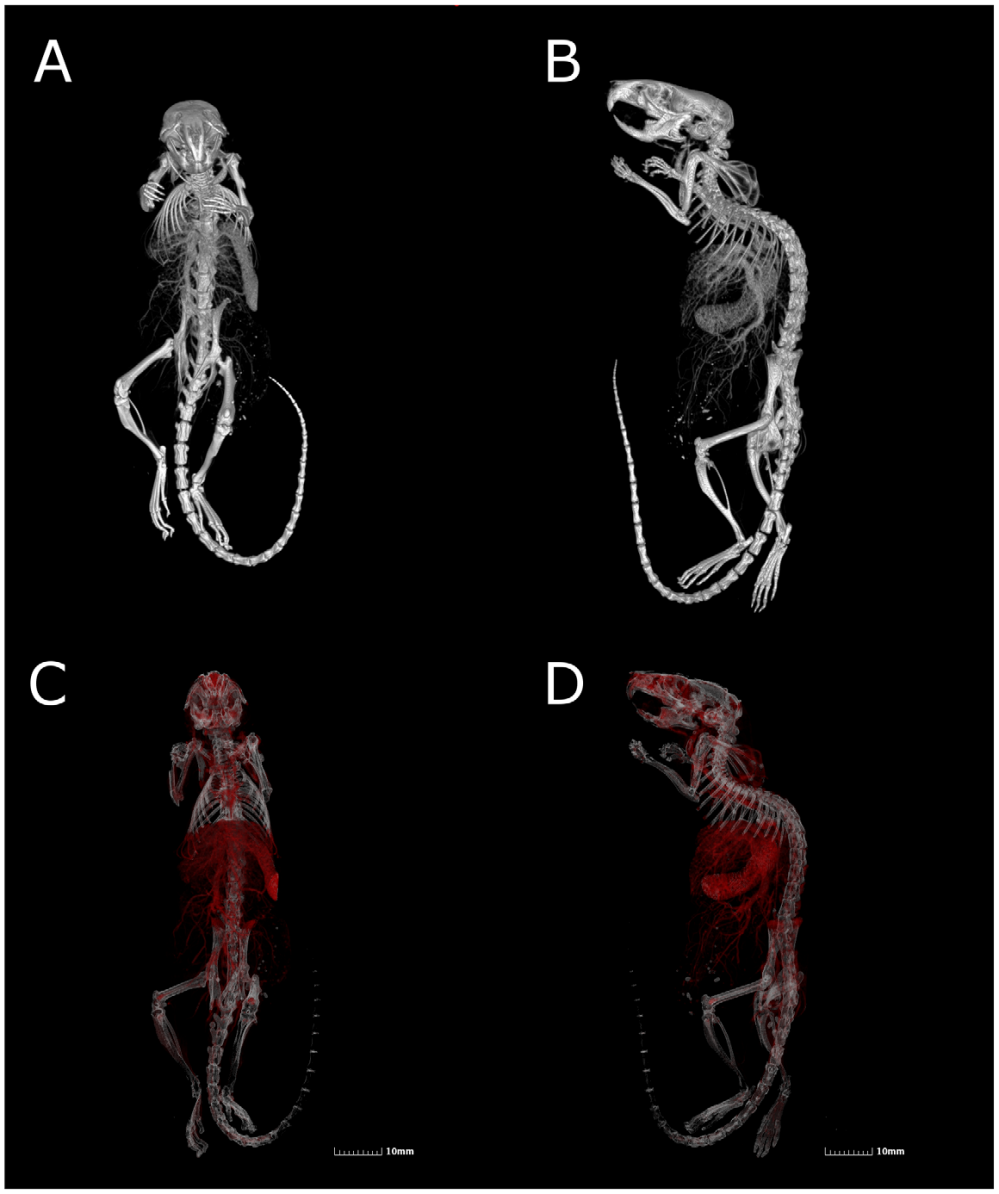
Intact whole-mount specimen vasculature imaging is possible by µCT using radiopaque silicon polymer medium perfused animals. All panel images derived from the same Microfil perfused adult mouse data set. (A–B) Volume rendered grayscale images of skeleton and perfused vasculature. (C–D) Volume rendered pseudocolored images of perfused vasculature (red) and bone (white).

With postmortem MicroFil perfusion and *ex-vivo* analysis, the entire blood volume can be imaged in-place. In Figure 3, we highlight whole mount specimen perfusion and show how associated grayscale values of the perfusion medium can be used to create high-impact rendered images. In Panels A and B, grayscale MIP images were rendered to highlight perfused thoracic and abdominal vasculature of an entire adult mouse specimen. Panels C and D include pseudocolored regions of vasculature based on grayscale threshold values. The red coloration allows for more subtle areas of vasculature to stand out against background.

### MicroFil image data can be rendered to display quantitative vessel morphometrics in a variety of perfused organ vascular regions

**Figure 4:**
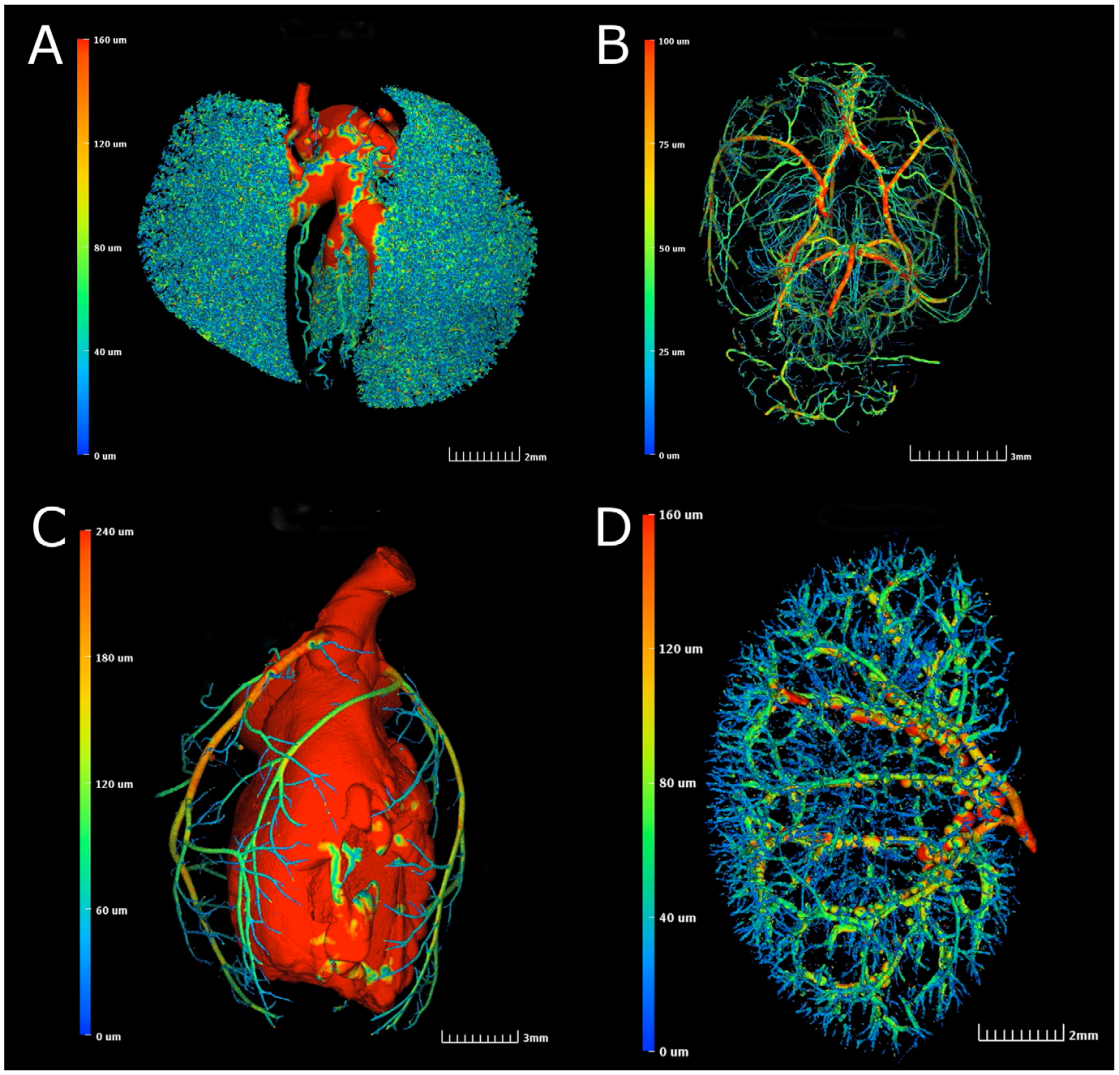
Vessel morphometrics can be derived from perfused mouse organ vasculature. Color bar on left of each panel indicates associated vessel diameters. (A) Pseudocolored image of adult mouse lung vasculature. (B) Pseudocolored image of volume rendered coronary vasculature of an adult mouse brain. (C) Pseudocolored image of volume rendered vasculature of an adult mouse heart. (D) Pseudocolored image of volume rendered vasculature of an adult mouse kidney.

In order to gain insight into the vessel morphometry associated with perfused organ vasculature, we computed a 3D skeleton for the segmented tissue using the technique of Lee, *et al*. [4]. For each point on the skeleton, we use simple ray casting to compute the average diameter of the vessel in the direction of the normal plane (Figure 4). The generated color scale on the left-hand side of each panel is associated with the colors observed in the volume-rendered organs. A given color represents a numerical value indicative of the cross-sectional vessel diameter through each region of vasculature. In this way, it is possible for an observer to quantify regional vessel diameter changes at a glance and throughout the entire organ volume.

## Discussion

In this paper, it is shown that microCT imaging of Microfil perfused vasculature is sufficient to visualize and quantify systemic vasculature in preclinical mouse specimens. In addition to the three spatial dimensions afforded the observer, volume data can be rendered to derive further qualitative analysis. It is also shown that perfused specimen vasculature can be imaged whole-mount and digitally disambiguated from skeletal background. Lastly, it was demonstrated that isolated mouse specimen organs could be rendered to derive quantitative metrics and pseudocolored to highlight the main vascular regions of interest. Observed drawbacks to the method include incomplete vessel filling or vessel rupture, but can be minimized by amending the perfusion mass volume according to the average physiological blood volume of the laboratory mouse [5]. By moderating perfusion pressure and perfusion mass volume, individual investigators may find that further optimization is needed to amend the protocols depicted here for their particular preclinical model specimen. Additionally, the Microfil perfusion mass can be modified to increase or decrease the working time (i.e. time before hardening), and viscosity, as per the manufacturer's instructions.

Systemic blood visualization is generally performed *in-vivo* within focal acquisition parameters restricted to a limited field of view for therapeutic diagnosis [6],[7] although advances in angiography using synchrotron radiation have shown promise in increasing field of view while simultaneously increasing spatial resolution [8]. Further, the rhythmic movements of the heart and lungs can act as artifact inducers in tomographic reconstruction, limiting effective resolution and negatively impacting analysis, although efforts to correct these artifacts is actively being investigated [9],[10]. Additionally, the metabolic half-life of vascular perfusion reagents is short, limited to several hours or days for both magnetic resonance [11] and µCT studies [12].

Indeed, several experiments have been conducted using cast perfusion mediums to identify gross regional vascular anatomy *ex-vivo* [13],[14]. Additionally, tomographic studies utilizing microCT and Microfil perfusion mediums were able to derive volumetric [15] and pathological data [16]. By the methods detailed in this study, additional complement is provided to the analytical repertoire available to microCT technologists studying preclinical rodent vasculature.
